# How does culture affect experiential training feedback in exported Canadian health professional curricula?

**DOI:** 10.5116/ijme.58ba.7c68

**Published:** 2017-03-17

**Authors:** Kerry Wilbur, Rasha Mousa Bacha, Somaia Abdelaziz

**Affiliations:** 1 College of Pharmacy, Qatar University, Doha, Qatar

**Keywords:** International medical education, feedback, culture, Canadian, health professional, training, Qatar

## Abstract

**Objectives:**

To explore feedback processes of Western-based health professional student training curricula conducted in an Arab clinical teaching setting.

**Methods:**

This qualitative study employed document analysis of in-training evaluation reports (ITERs) used by Canadian nursing, pharmacy, respiratory therapy, paramedic, dental hygiene, and pharmacy technician programs established in Qatar. Six experiential training program coordinators were interviewed between February and May 2016 to explore how national cultural differences are perceived to affect feedback processes between students and clinical supervisors. Interviews were recorded, transcribed, and coded according to a priori cultural themes.

**Results:**

Document analysis found all programs’ ITERs outlined competency items for students to achieve. Clinical supervisors choose a response option corresponding to their judgment of student performance and may provide additional written feedback in spaces provided. Only one program required formal face-to-face feedback exchange between students and clinical supervisors. Experiential training program coordinators identified that no ITER was expressly culturally adapted, although in some instances, modifications were made for differences in scopes of practice between Canada and Qatar.  Power distance was recognized by all coordinators who also identified both student and supervisor reluctance to document potentially negative feedback in ITERs. Instances of collectivism were described as more lenient student assessment by clinical supervisors of the same cultural background. Uncertainty avoidance did not appear to impact feedback processes.

**Conclusions:**

Our findings suggest that differences in specific cultural dimensions between Qatar and Canada have implications on the feedback process in experiential training which may be addressed through simple measures to accommodate communication preferences.

## Introduction

The deepening intimacy of the global health community is marked by an expanding multicultural workforce, ongoing investment in international student and faculty exchange, and increasingly, the export of Western-based health professional curricula.^1^^,^^2^ A growing number of universities with health faculties of repute are establishing affiliate or satellite programs abroad permitting place-bound host country students access to high caliber training and exporting organizations the opportunity to promote their educational brand into new, and often resource-generating, markets.^3^ Canadian institutions are engaged in such partnerships in the Middle East country of Qatar where branch campuses have been introduced and program accreditation pursued as the nation commits to complementary initiatives towards improving both education and health care infrastructures for its burgeoning population.^4^^,^^5^ Diploma and degree nurses have been trained at the University of Calgary-Qatar (UC-Q) since 2006.  Newfoundland and Labrador’s College of the North Atlantic has offered degrees in respiratory therapy, emergency medical services, dental hygiene, medical radiography, and pharmacy (technicians) for over a decade through its local campus (CNA-Q). The curriculum at College of Pharmacy (CPH) at Qatar University (QU) is Canadian in conceit and delivery and accredited by the Canadian Council for Accreditation of Pharmacy Programs (CCAPP).  Although undergraduate medical education is presently delivered by New York’s Weill-Cornell Medical College in Qatar (WCMC-Q), the Royal College of Physician and Surgeons of Canada (RCPSC) Office of Specialty Education has been commissioned by the country’s health ministry (Supreme Council of Health) to develop and implement the first national continuing medical education and accreditation standards for all health practitioners. In this way, Canada is further extending its expertise in medical education and professional learning in Qatar.^6^^,^^7^

While enthusiastically received, cross-border partnerships are not without challenges.  Host country health care systems may not easily or be entirely capable to accommodate instruction and practice of all outlined professional competencies and local faculty may be more accustomed to conventional teaching methods.^8^^,^^9^ Exported curricula may be subject to reservations within the home country associated with the capability to uphold the indigenous program’s rigour and reputation overseas.^10^ Such issues may impact all aspects of the intended curriculum, including practice-based learning. 

Experiential training (known in some countries and disciplines as “clinical apprenticeship”, “clerkship” or “workplace-based training”) is an integral component to health professional programs in that students have the opportunity to consolidate knowledge and skills under the mentorship of clinical supervisors or “preceptors” in authentic care settings.^11^ These clinicians are often also responsible for the completion of in-training evaluation reports (ITERs) whereby performance is directly observed and the assessment of the student’s relative strengths and weaknesses is recorded.^12^ However, caution must be exercised when curriculums moving across borders also export assessment tools and processes to new environments without accounting for local perspectives.  While feedback processes are recognized in the promotion of learning, verbal and written feedback exchange between students and supervisors may be subject to differences in cultural norms and values.^13^^,^^14^

Culture has been defined as the sharing of a collective identity, common history and experience, and shared beliefs, values, and norms.^15^ Hofstede has put forth a theoretical framework to describe a country’s cultural predisposition according to its population’s proclivity across six main domains (power distance, collectivism, uncertainty avoidance, long term orientation, indulgence and masculinity).^16^  This cultural dimensions’ concept has been previously employed in health research, notably as it pertains to understanding communication between health professional and patients among international medical graduates joining practice in the UK.^17^^,^^18^ According to Hofstede, relative distinctions between Canadian and Middle East regions exist in cultural dimensions of power distance, individualism and uncertainty avoidance.  Power distance is the extent to which members of a particular culture accept the inequalities of authority and may be reflected in the reverence offered to such figures.  Qatar society is characterized by higher power distance when compared to Canada and this faith in hierarchal order may have greater influence on students’ comfort and ability to offer constructive criticism to those in supervisory roles during their experiential training. Members of an individualist culture (Canada) emphasize independence and the achievement of personal goals, whereas those from a collectivist culture (Qatar) tend to prioritize group loyalty and value interdependence.  Such collectivist features may have implications similar to high power distance in feedback exchanges and may additionally affect supervisor evaluations when more than one student is concurrently mentored during the same internship experience (distinct feedback is not provided to students).  Finally, uncertainty avoidance relates to how a culture deals with ambiguity.  Some societies, like in Qatar, create and ascribe to rigid infrastructures, whereas in Canada there exists greater tolerance for unorthodox behavior or ideas and is relatively less rules-oriented.  Members of cultures where uncertainty avoidance is high may not openly engage in feedback systems unless they are absolutely certain of a favourable outcome.^19^ Meanwhile, Edward Hall has delineated cultures according to their use of context and information to create meaning.^20^ For example, a high context society like Qatar may rely on nuanced and non-verbal communication in contrast to Canada where messages are generally transmitted explicitly.  Processes of direct feedback may be interpreted as overly critical in a high context society and may challenge a program’s ability to acquire documented quality constructive narrative components as part of a Canadian-generated ITER.^21^

Widely accepted feedback traditions in Canadian education systems may not be readily or effectively integrated in to foreign settings.  Feedback preferences and behaviours have been widely explored within various business-oriented industries, but its study within medical education is largely lacking.^22^   We sought to determine the experiential training feedback processes of Canadian health professional programs established in Qatar and explore program coordinator perceptions of how these may be influenced by the cultural context.  

## Methods

### Study design

This qualitative study employed document analysis and semi-structure interview. Document analysis is systematic procedure for identifying, reviewing, and deriving useful information and understanding from existing documents.^23^ All Canadian health professional programs training students in Qatar were contacted to acquire hardcopies of ITER forms used by clinical supervisors to assess students and any whereby students evaluate the clinical supervisor or site. The static characterization of item and feedback features and processes for their use in the respective health professional programs was further explored by key informant interviews.

**Table 1:** Canadian health professional experiential feedback processes in Qatar

Program	Nursing	Pharmacy			Dental Hygiene	Paramedic		Pharmacy Technician	Respiratory Therapy	
Campus	University of Calgary, Qatar				Qatar University			College of the North Atlantic, Qatar		
Accreditation	CASN*		CCAPP^†^		Not-accredited follows CDAC^‡^		CMA^¶^ pending	CCAPP	CoARTE^**^	
Rotation Length	328 hours		4 weeks each				12 weeks			
Clinical Supervisor Feedback To Student										
Competency Evaluation	two response items: ‘satisfactory’ and ‘unsatisfactory’		three response items: ‘above’ or ‘meets expectations’ and ‘needs improvement’		two response items: ‘complete’ and ‘incomplete’		two response items: ‘‘complete’ and ‘incomplete’	three response items: ‘excellent’; ‘satisfactory’ and ‘needs improvement’		two response items: ‘complete’ and ‘incomplete’
Timing	midpoint & final		midpoint & final		weekly		weekly	midpoint & final		weekly
Mode	electronic submission				written hard-copy			electronic submission		
	asynchronous				face-to-face			asynchronous		
Student Access	rotation-end by checking electronic system		real time		rotation-end by checking electronic system					
Student Feedback To Clinical Supervisor/Site										
Timing	course-end		rotation-end	rotation-end			semester-end	rotation-end		semester-end
Mode	electronic submission			written hard-copy				electronic submission		
	asynchronous blinded			face-to-face				asynchronous blinded		
Method	not mandatory		uniquely each rotation	uniquely each rotation			1 question in larger student survey	uniquely each rotation		1 question in larger student survey

*CASN = Canadian Association of Schools of Nursing; †CCAPP= Canadian Council for Accrediting Pharmacy Programs; ‡CDAC = Commission on Dental Accreditation Canada; ¶CMA = Canadian Medical Association; **CoARTE = Council on Accreditation for Respiratory Therapy Education

### Study participants and recruitment

The study participants included the experiential coordinators of the six health professional programs delivering Canadian curricula in Qatar:  UC-Q nursing, QU CPH pharmacy, and CNA-Q respiratory therapy, emergency medical services, dental hygiene, medical radiography and pharmacy technician programs.  Individuals were first contacted by email whereby the study and its objectives were summarized.  Experiential coordinators expressing interest in participation were then contacted by telephone to confirm their participation and schedule an interview at their place of work.  The consent form was then sent by email for their review and signed when the interviews took place.

### Key informant interviews

Semi-structured interviews were conducted in person following a question topic guide informed by Hofstede and Hall’s cultural theoretical frameworks (see Appendix).^16^^,^^20^ The interviewer could rephrase the questions or elaborate upon its intended meaning as deemed necessary. Participants could speak to any question for as long as they wanted as time was not expressly restricted.

### Data analysis

All instruments used as ITERs by clinical supervisors to assess students in the health professional programs, as well as any form students submit in turn as evaluation of their clinical supervisor were described and categorized. The semi-structured interview discussions were audio-recorded and transcribed verbatim by one researcher and then verified by a second researcher attending the interview.  Finalized interview transcripts were evaluated independently by two researchers using directed content analysis whereby various portions of the transcribed data were divided into units at the level of the phrase, sentence, or paragraph and examined for ideas and messages.^24^ The coding process and theme assignment were derived from the cultural frameworks of power distance, collectivism, uncertainty avoidance, and context selected a priori, but additional conceptual categories were explored.  To further validate our interpretations, summaries of each verbal exchange were returned to the corresponding participant to determine if they accurately reflected his or her response’s original intent or meaning. Ethics approval was obtained by QU Institutional Review Board.

## Results

### Document analysis

Seven documents were obtained from these six programs and are summarized in Table 1. All programs had obtained or were seeking Canadian-accreditation. Students participated in experiential training in Qatar workplaces supervised by both local preceptors and faculty clinical instructors. The internship duration was variable, ranging from isolated days each week throughout the semester to 4-12 week devoted blocks of full-time workplace training, with most occurring in senior years of the programs.  In some disciplines, multiple members formally participated in student teaching during any given internship (respiratory therapy, dental hygiene) whereas in others students were matched with few for the duration (emergency medical services, pharmacy).  All program ITERs outlined competency items for student’s to achieve by the end of the internship period.  Clinical supervisors choose a response option corresponding to their judgment of student performance and may provide additional written feedback in spaces provided. No ITER was expressly culturally adapted, although modifications were made when differences in local scopes of practice were detected (for example, tasks associated with pharmacy technicians handling of narcotics were removed).  While all encouraged daily verbal formative assessment, at least one program required formal clinical supervisor documentation of student performance on a daily basis (paramedics).  Nursing and all CNA-Q programs utilized an electronic system for asynchronous student and experiential program coordinator review while pharmacy ITERs were completed by hand for submission. Pharmacy preceptors gave written and verbal evaluations to students in an internship exit meeting whereby these documents were signed by both parties before faxed or emailed to the experiential program coordinator. Students in pharmacy also offered in-person written and verbal feedback to clinical supervisors at the internship’s conclusion; those in all other programs instead submitted a semester-end course survey which included one question related to their clinical training site.  Student self-assessment or reflection documentation was completed all programs.

### Key informant interviews

Several examples of cultural impact on feedback processes were given in the interviews with the 5 Canadian and 1 American experiential program coordinators.

### Power distance

All coordinators described issues arising that were consistent with of power distance effects whereby students were unwilling to give constructive criticism to clinical supervisors.

“Some students are shy and are afraid to give feedback because it might get them in trouble.” (Participant 3)

“The other thing is the authority, especially if the student is Qatari and the preceptor is non-Qatari. Sometimes they think if this is a Qatari student and if I do not say good things, I will be in trouble” (Participant 5)

“Students might be hesitant to report some issues and when they come later with preceptor concerns [they do so] without naming names because they will have to work with them after graduating” (Participant 2)

Once anonymous means were made available in some programs, such as blinded (to the preceptor) submission in an electronic system, the veracity of such feedback was felt by coordinators to have increased. Paradoxically, availability of such blinded feedback mechanism was often countered with student reluctance to record supervisor or site recommendations on these ITERs (low context).  Coordinators of all programs perceived student and clinical supervisor preference to interact in-person with the faculty clinical instructors or experiential program coordinators to share potentially negative information or experiences. 

“[Students] are happy to speak to [coordinators] but less comfortable documenting it…because of the perception that documenting makes it more permanent” (Participant 1)

“I suspect [supervisors] will wait over repeated attempts before rating achievement; they are not going to document each time the student fails the competency before the end”  (Participant 5)

### Collectivism

A few instances could be attributed to greater collectivism in Qatar than in Canada. Discrimination in performance when paired students were sharing an internship supervisor was sometimes not discernable when submitted ITERs are compared.

“For the most part, the comments are similar when there are more than one student at a site. Unless the student is very poor, then I do see differing grades and comments” (Participant 6)

One coordinator also described occasions when unsatisfactory student behavior (e.g. absenteeism) was recorded by clinical supervisors, which would be contradicted in reports by other preceptors who shared the student’s ethic background.

“We had one [Arab] student only showing up in the morning. Feedback from Arab preceptors was that he was there, but his other [Asian] preceptors told us that he was not” (Participant 5)

### Uncertainty avoidance

No participant could offer any example of how differences in uncertainty avoidance might tangibly affect feedback in this setting.

## Discussion

If we consider experiential training fundamental to health professional education, then feedback is central to the enterprise. Clinical supervisors often have dual roles to facilitate learning opportunities and role model care in the internship setting, as well as advise students how they might improve their performance.^25^ Ideally, the provision of such information will motivate students to make adjustments and continuously improve their level of competency.  In turn, students’ ability to offer feedback about clinical supervisors and sites can identify positive ways learning has been supported, but also alert program coordinators to sub-optimal conditions requiring attention.^26^^,^^27^ However, many factors influence the utility of workplace-based student feedback, including credibility of the source and legitimacy of the content in terms of adequacy of observations or the relationship with the assessor.^28^^-^^30^ While the study of feedback processes in medical education is considerable, it is far from exhausted.  To date, very little investigation of exported health professional curriculums has been performed and specifically, exploration of feedback processes within the experiential training component in these diverse cultural contexts.8,10 Extensive research in organizational psychology has shown how different cultural value systems affect individuals’ perception of received feedback, shape feedback-seeking behaviours and how this in turn influences the workplace and its management.^31^ Specific strategies have been put forth to address feedback in culturally diverse settings, such as matching feedback to both team and individual goals (collectivism) and scheduling regular feedback meetings (uncertainty avoidance).^31^

Our findings illustrate various approaches to experiential feedback among the different disciplines with established Canadian curricula in Qatar. Different measures of achievement for student competencies were outlined (as levels or dichotomous ratings), as well as expectations and formal mechanism for students to evaluate their clinical supervisors.  Review of ITER documents and discussion with experiential program coordinators underscore how the quantity and veracity of feedback may be affected by the medium of exchange.  In the program continuing to use paper-based ITER completion (pharmacy), especially when in-person “360 degree” feedback between students and clinical supervisors was expected, experiential program coordinators described a disconnect between what they read in the submitted evaluations and what they learned separately from students (and clinical supervisors) when given an opportunity. When there is anonymous or indirect evaluation, the candour of both student and supervisor commentary increases and prior evidence of power distance is essentially eliminated. Programs at CNA-Q use an electronic system akin to ONE45 used for medical resident evaluation throughout Canada, whereby ITERs are completed by clinical supervisors online. Additionally, many CNA-Q program ITERs have a relatively higher proportion of technical competencies as part of the experiential training (such as performing compounding calculations (pharmacy technicians) or removing endotracheal tube (respiratory therapy)) when compared to nursing or pharmacy and as such, student assessment was considered less subject to cultural influences by the experiential program coordinators interviewed.

That the student’s or supervisor’s true perspective is not documented in writing would be consistent with preferences of a low-context culture, such as Qatar. However, obtaining quality narrative is not exclusive to this Arab setting. Study of the written record to support assigned scoring on Canadian medical resident ITERs has shown gaps in the quality of feedback, which may be improved with directed faculty development program.^21^^,^^32^ In cases of poor performance especially, Canadian physicians have identified a kind reluctance to document slightly negative feedback for fear of a disproportionate effect on the medical student’s or resident’s future career opportunities.^33^ Others have explored how Canadian faculty attendings infer intended meaning by “language cues” found within ITER narratives.^34^ Our research question also incorporated the students’ assessment of the clinical supervisor which has been less well-described; however recent study of physiatrists residents outlined challenges of in-person open feedback including discomfort offering constructive comments and concerns for negatively influencing their relationship with the supervising staff physician resulting in the perceived need to soften corrective feedback messages.^35^

We could not delineate many instances of how differences in cultural dimensions of individualism and uncertainty avoidance reported between Canada and Qatar might impact feedback. Lack of expected group-oriented evaluations in this collectivist environment could in part be attributed to the discrete nature of technical competencies evaluated coupled with the low number of paired student or supervisor compositions among most of the programs (that is few 2 students to 1 supervisor ratio, or vice versa). Literature seems lacking on this phenomenon within health disciplines, particularly study of how physician supervisors might differentiate assessment among medical students or residents who often train concurrently in the medical model. Inquiry has been into how in the opportunistic environment of patient care in experiential learning, clinical supervisors assign tasks to different levels of learners, but not subsequent evaluation.Members of cultures high on uncertainty avoidance are risk averse and prefer predictable environments. While we hypothesized that students and clinical supervisors in Qatar would therefore eschew processes and circumstances whereby negative feedback might be exchanged, it is possible that the structured mechanisms delineated by each health professional program were instead accepted as rules for the group norm and so members complied with its expectations.

A number of limitations to this exploratory study merit consideration. We relied heavily on Hofstede’s cultural theoretical framework mapping dimensions of a nationality’s preferences, but must acknowledge, as he does, that individual variations exist. While we have described Canadian and Arab cultural features and associated behaviours as monolithic, not all members of a population strictly ascribe to its outlined model. Our research question presupposed that the Arab students would be supervised by local supervisors of Arab-origin and we did not previously take into account the number of Canadian clinical faculty members that we discovered were embedded within care settings as instructors. It is unknown if their feedback behaviours would differ from local supervisors and how this would subsequently affect experiential program coordinators overall impressions. Similarly, all coordinators interviewed were of North American-origin and may perceive cultural aspects of feedback through a different lens. Full appreciation of feedback processes is limited by document analysis and experiential program coordinators interview alone and we intend to conduct further study with the students and clinical supervisors of these programs.  Finally, our research question did not incorporate inquiry into student reflection or self-assessment, which forms an important component of many health professional experiential training curricula in Canada. Although Canadian physician training has not yet taken foothold in the Middle East, overseas partnerships are in place elsewhere and understanding of how medical student and resident feedback is considered, even in seemingly like-minded cultures, is essential for quality assurance.^37^^,^^38^ While our findings did not uncover distinct feedback as perceived by the experiential coordinators of Canadian programs in Qatar, full appreciation of feedback processes is limited by document analysis and experiential program coordinators interview alone and we intend to conduct further study with the students and clinical supervisors of these programs.

Our results have implications within this Arab context whereby existing health professional programs would benefit from purposeful revision of ITER format and documentation, but also for Western-oriented curricula entering the regional market to educate these and students of other health disciplines (i.e. physiotherapy, dentistry, medicine) in the future.  For example, preferences specific feedback process in experiential training may be accommodated when anonymous and electronic systems are in place.  Health professional programs engaging in cross-border partnerships within Asian and African contexts may also inform cultural adaptation of experiential training feedback processes from our experiences. Finally, this research may also contribute to understanding of feedback preferences of foreign health professional students or graduates entering Western-oriented education and care settings.^39^

## Conclusions

We have explored how power distance, collectivism, uncertainty avoidance and cultural contexts might influence communication between clinical supervisors and health professional students in Qatar.  Our findings suggest that differences between Canadian and Middle East cultural dimensions have implications on the feedback process in experiential training which may be addressed through simple measures to accommodate communication preferences.

### Acknowledgments

This study was funded by a Qatar University internal study grant.

### Conflicts of Interest

The authors declare that they have no conflict of interest.

## Supplementary material

Supplementary file 1 Appendix. Semi-structured interview guide
