# Supplementary material for: How does culture affect experiential training feedback in exported Canadian health professional curricula?
Source: Int J Med Educ. 2017 Mar 17;8:91–8. doi: 10.5116/ijme.58ba.7c68 (PMC5376492; doi:10.5116/ijme.58ba.7c68)
Supplement: Supplementary file 1 — Appendix. Semi-structured interview guide [file ijme-8-91.S1.pdf]

Appendix

Semi-structured interview guide

1. Can you tell us more about the forms your program uses in the evaluation of your students during experiential training (or “clerkship” or “rotations”)? What is their source and have they been altered in any way for use in Qatar?

2. What is the feedback process between students and clinical supervisors in your program?

3. What is the nature of the written feedback? Is it sufficient for you to link student performance with the submitted evaluation form?

4. Do you believe the evaluations and feedback therein always reflect reality? If not, why not?

5. Can you think of any instances where you believed students were reluctant to offer authentic preceptor feedback? Can you think of any instances where you believed clinical supervisors were reluctant to offer authentic student feedback?

6. Why do you believe these situations may have occurred?

7. Could you attribute such situations to differences in culture, such as discomfort in challenging authority (explain power distance) or distinguishing individual performance (explain collectivism); desire to minimize uncertainty associated with feedback repercussions (explain uncertainty avoidance) or documentation of perceived criticism (explain high context communication)?

8. Have you developed any strategies to overcome challenges in your feedback processes? What has been the outcome?
